# The Book World of Medicine and Science

**Published:** 1900-04-21

**Authors:** 


### The Book World of Medicine and Science.

The Evolution of Asylum Architecture. By R. H. Steen, M.D. Lond. (Bartholomew Close, E.C.: Adlard and Son. Pp. 23 and plans.)

When the modern treatment of insanity sprang into prominence it did not take long for asylum medical officers to discover that their efforts at improvement were sorely cramped by the architectural blemishes of the buildings, and many attempts were made to remedy the more glaring defects. So far as we are aware, Holland, of Prestwich, was the first English medical superintendent who earnestly set to work to design a new asylum, and the fine structure at Whittingham was the result of his efforts. It was the best large asylum of its time, and in its main features it may still challenge comparison with the more recent ones. Since that time Wallis, of Hull, and Greene, of Northampton, have left their marks on the asylums of to-day. Dr. Steen's pamphlet is an eminently suggestive and instructive one, and it shows that much attention has been given to a difficult subject, and one not necessarily coming under his own province, hence greater praise is due. We are in full accord with the author in his remarks on the danger of separating the acute from the chronic patients. There is also much in what he says about the position of the chapel and of the medical superintendent's house. The vast majority of our English asylums have these in faulty positions. The chapel should invariably be attached to the asylum, and the superintendent's house invariably detached from it. Dr. Steen also correctly sums up against a common dining hall. The cruelty of sending patients long distances to their meals is wholly unnecessary, and yet it is done in more than half of our asylums. We are not sure that we can wholly approve of the model hospital or infirm block which Dr. Steen gives us, either as to all of its arrangements or to its position. For instance, there is not one of the single-bedded rooms which faces south, although patients might have to occupy these rooms for months at a time. We should like to have had Dr. Steen's views as to the heavy sums which our county asylums are now costing. He states that the Alt-Sherbitz Asylum cost £143 a bed inclusive of the site. We doubt whether it could be done in England for that sum, although it is believed to be a model of its kind. The pamphlet includes plans of the asylums at Chichester, Woodielee, Derby, Bexley Heath, Gartlock, and Alt-Sherbitz.

Health Abroad: A Medical Handbook of Travel. Edited by Edmund Hobhouse, M.D. (London: Smith, Elder, and Co. 1899. Price 6s.)

The object with which this book has been compiled is to enable the ordinary traveller to preserve his health whilst travelling in unknown, or at least in unaccustomed climates, and to make him familiar with the various emergencies which may arise, so as to be able to treat them to the best advantage when medical aid is not forthcoming. The authors of all the articles which it contains have, it is stated, had personal experience of their subjects, and this is borne out by the list of contributors. On turning to its pages one soon sees that the book contains a large amount of information of a geographical and descriptive character which is only incidentally connected with the more purely health or medical side of the questions dealt with, and these details about climate in various parts of the world are really very interesting, while the descriptions are in some places not without a certain literary merit. It may be well to state that the book deals with all sorts of countries, and that there is none of that exclusive attention to tropical regions which makes some books on the subject read as if all foreign countries were hot. Scattered throughout it are many hints as to the treatment of the various diseases which are apt to be met with in the several places described, and at the end are short chapters on the treatment of wounds and on diphtheria. But there is throughout a complete absence of quackery or of any pretence that by its aid people can get on without the doctor. The directions which are given in regard to treatment are mostly confined to what it is possible for the traveller to do for himself or his companion when no professional assistance can be obtained. But with the object of showing where such help can be got, we note that the local list of registered medical practitioners resident abroad, extracted by permission from Churchill's "Medical Directory," is given as an appendix at the end of the book. The book is likely to be useful to the traveller, and it certainly is interesting to the stay-at-home reader as telling many details of many climes.

Brain and Body; The Nervous System in Social Life. By Dr. Andrew Wilson. (London: James Bowden. 1900. Price 1s. 6d.)

This is a pretty little book, whose author has a pretty little knack of absorbing scientific knowledge from various sources and emitting it again in popular guise. He is an admirable example of the modern scientific go between, on whom we nowadays depend for ready-made knowledge; something more than a mere purveyor of mental food; rather a cook, who by his art makes scientific soufflés out of the solid stuff provided in the ordinary manuals of science, and thus fits it for the fickle appetite of a public which wants to know everything but does not want to learn. So far as it goes this elegant introduction to nervous physiology is very good. It certainly is very readable, and it flows on in that easy-going style which soon induces in the reader the idea that he knows all about it. What more can we say? We begin with a chapter on "the bashful man," in which this blushing creature is made the centre of an essay on the nervous system; then we are led on to a description of reflex action, and from reflex blushing we come easily to the nervous mechanism of the heart; and so to memory and mind, and body and diet, and alcohol and tea, and tobacco and all the rest of it. It is all very good, and we have no doubt that the author could have easily run on for ten volumes of the same character had not his publisher put the limit at the 142 narrow pages now before us. Our only objection to books of this sort is that they produce a breed of unpleasant and inquisitive patients who cause an unconscionable waste of the doctor's time. That, however, is a trifle, and against it may be placed the consoling fact—consoling, that is, from the professional point of view—that at any rate such books, and such tasty bits of lightly-cooked knowledge, make people fidgetty about themselves and drive them to our consulting rooms, and with income-tax at a shilling we tender our grateful thanks to anyone who will send us a few patients.

